# Tai Chi Can Improve Postural Stability as Measured by Resistance to Perturbation Related to Upper Limb Movement among Healthy Older Adults

**DOI:** 10.1155/2016/9710941

**Published:** 2016-11-30

**Authors:** Jiahao Pan, Cuixian Liu, Shuqi Zhang, Li Li

**Affiliations:** ^1^Key Laboratory of Exercise and Health Sciences of Ministry of Education, Shanghai University of Sport, Shanghai 200438, China; ^2^Kinesiology and Physical Education Department, Northern Illinois University, Dekalb, IL 60115, USA; ^3^School of Health & Kinesiology, Georgia Southern University, Statesboro, GA 30460, USA

## Abstract

*Purpose*. The aim of the study was to examine the effects of Tai Chi (TC) training on postural control when upright standing was perturbed by upper limb movement.* Methods*. Three groups, TC, Brisk walk (BW), and sedentary (SE), of thirty-six participants aged from 65 to 75 years were recruited from local community centers. Participants performed static balance task (quiet standing for 30 s with eyes open and closed) and fitting task (two different reaching distances X three different opening sizes to fit objects through). During tasks, the COP data was recorded while standing on the force plate. Criteria measures calculated from COP data were the maximum displacement in anterior-posterior (AP) and medial-lateral (ML) directions, the 95% confidence ellipse area (95% area), and the mean velocity.* Results*. No significant effect was observed in the static balance task. For fitting tasks, the group effect was observed in all directions on COP 95% area (*p* < 0.05) and the TC group showed reduced area. The tests of subject contrasts showed significant trends for reaching different distances and fitting different openings conditions in all directions, the 95% area, and the mean velocity (*p* < 0.05).* Conclusion*. Compared to the other two groups, long-term TC exercise helps in reducing the effects of upper body perturbation as measured by posture sway.

## 1. Introduction

Postural control is the ability to control the body's position and is important for daily living activities [1]. When performing daily activities, such as walking, talking, and cleaning [2], people mostly maintain upright posture. Postural control ability decreases with ageing [3, 4]. For example, Prieto and colleagues observed that center of pressure (COP) movement decreased in increments of 3 to 5 years in the young adult group but increased in the elderly group [4]. Furthermore, postural control has been more relevant to the risk of falling in older adults as compared to younger people since the elderly experience elevated fall risks [5, 6].

Therefore, decreasing postural stability presents a serious challenge to elderly people with increasing risk of falls. By the middle of 21st century, China will become the country with mostly elderly population [7]. In 2011, the Chinese Health Organization reported that, compared with other major epidemics, the highest mortality rate correlated to falls for people 65 years and older, 49.65/100 thousands for men and 52.80/100 thousands for women (Chinese Health Organization projections, 2011). The most common fall-related injuries include abrasions, open wounds, fractures, and brain damage [8]. Although no official document reported annual medical costs directly associated with falls in China, it should be very high on an estimation with no doubt. The data reported in the literature that spending was nearly 20 billion dollars in the US [9].

Falls most likely occur when elderly people engage in multitask activities [2]. Upright stance with an additional concurrent task that could be associated with motor, sensory, or cognitive function leads to the increased the risk of falls. Standing with upper body movement (grabbing, reaching, fitting, etc.) is an important activity in daily life which is closely associated with fall risks among elderly people [10]. Ten years of age appears to be the transition period when children have greater adaptability and reach a degree of freedom similar to adults when performing standing with upper body movement [11]. No significant difference is observed in COP patterns between 10-year-olds and adults [11, 12]. However, the ability of postural control appears to develop, be maintained, and decline throughout life [13]. Consequently, standing with upper body movements may contribute to reduced stability, mobility, and quality of life in elderly people. Overstall and coworkers have demonstrated that rapid arm movements may induce falls in the older adults [14].

Physical activity is an effective strategy for improving postural control and decreasing fall risks among the elderly. For example, Brisk walk (BW) is a cyclic and aerobic exercise which could improve mobility, strength, and endurance [15, 16]. Walking can improve triceps surae muscle strength [15] and improve maximal oxygen uptake (*V*_O2_ max) [16] for ageing populations. However, research demonstrates that although elderly walking group showed significantly better postural stability during static conditions, no difference in postural limit test was observed comparing to nonwalking group [17]. Postural limit test asks the participants to actively explore the boundaries of their own postural control capacity which is very different from quiet standing where stability boundaries are not challenged.

Tai Chi (TC) is a traditional Chinese martial art which emphasizes slow and smooth movement accompanied with rhythmic weight shifting and limb coordination. During Tai Chi practice, stability boundaries are constantly challenged. Long-term TC exercise leads to a significantly smaller passive motion detection threshold than that observed in the SE comparison group [18]. Therefore, it could improve strength, proprioception, psychological well-being, and balance [18–22]. In addition, many researches also demonstrated the effectiveness of TC in reducing the risk of falls for elderly people [19, 20]. For example, Guan and Koceja observed that the postural sway of the TC group was less significant than the control group during standing [19]. For another example, Li and Manor indicated that TC exercise increased functional gait and leg strength performance among people with peripheral neuropathy [21].

Much research has demonstrated that postural control could be influenced by ageing [3, 4]. In contrast, physical activities improved posture stability [18, 22, 23]. Many studies reported better postural control after TC exercise in the elderly population. However, majority studies measured lower limb performances when evaluating balance abilities. In real world situations, the perturbation of upper body motion is a particularly challenging task for elderly people when maintaining postural stability. The purpose of this paper is to explain that (1) upper extremity motion affects postural stability measured through COP, (2) postural stability of both long-term exercise groups should be perturbed by upper body motion less than the control group, and (3) TC group should perform better than the BW group.

## 2. Materials and Methods

### 2.1. Participants

Thirty-six apparently healthy participants aged 65 to 75 years were recruited from local community centers using snowball method. They were accordingly classified into three groups: Tai Chi (TC), sedentary (SE), and Brisk walk (BW). The TC group regularly practiced Tai Chi and the BW group took regular Brisk walks for more than 5 years (more than 3 times per week and more than 1 hour at a time); the SE group did not participate in regular exercise (less than 1 hour of purposed exercise per week). The subjects signed the informed consent which was approved by the Institutional Review Board of the Shanghai University of Sport.

Exclusion criteria were (1) lower extremity and/or dominant arm/hand surgery; (2) cardiovascular pathologies, diabetes, or hepatorenal syndrome; (3) coordination function disorders such as peripheral neuritis, Meniere disease, Parkinson's disease, and Alzheimer's disease; and (4) BMI > 30.

### 2.2. Instrumentation Setup

**Figure 1:**
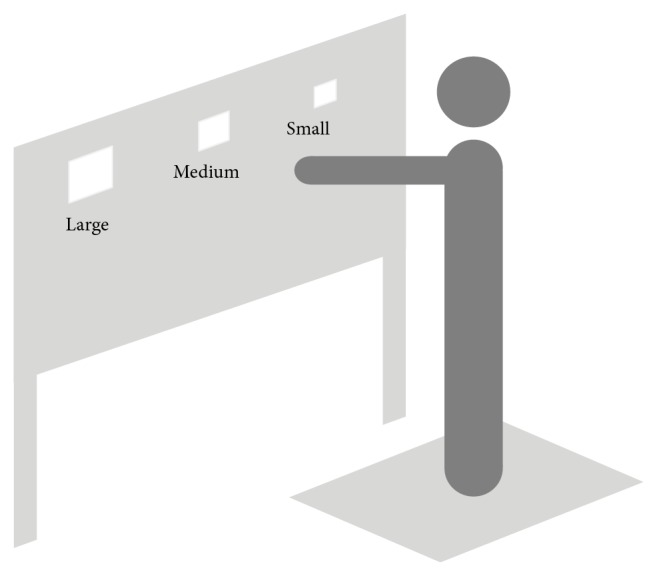
Experimental setup: instrumented fitting board and force platform.

A self-manufactured instrument was used in the study (illustrated in Figure 1). The instrument had a large object placement board (1200 *∗* 600 mm) that contained three side by side openings and a fixed space between each opening of 300 mm. The sizes of the openings were large (130 *∗* 130 mm), medium (115 *∗* 115 mm), and small (100 *∗* 100 mm), respectively. The board could be adjusted, according to the participant's shoulder height and arm length, and three optical gate sensors were attached to the back of the placement board and the upper edge of each opening. Another sensor was attached to the basement that supported the fitting block (90 *∗* 90 mm) on the table. A cylindrical handle with a length of 20 mm and diameter of 20 mm was attached to the block to allow for comfortable grasp. The sensors were used to record fitting time during the fitting task. Additionally, a recessed Kistler force plate (60 *∗* 90 cm) (Kistler 9287c, Kistler Corporation, Switzerland) was sampled at 1000 Hz to obtain the force data. The optical gate sensors and force plate were synchronized.

### 2.3. Testing Protocol

Participants performed two different tests including the static balance task (quiet standing) and the fitting task (standing with upper body movement). Prior to the data collection, height, mass, dominant arm length, and shoulder height were recorded for each participant. Through the entire tests, participants were wearing uniform socks.

In the static balance task, participants were required to stand at the center of the force plate with their feet forming a 30° angle and their heels 8% body height apart [24] for 30 s with eyes open (EO) and 30 s with eyes closed (EC). In the EO test, each participant was instructed to focus on a target positioned in the individual's line of vision at a distance of 3 meters. Each trial was repeated 3 times, and participants took a 2-minute break after each trail.

Prior to the fitting task, the experimental operator explained the test to each participant instructing them to align their middle line with the opening's center using the same foot position as in the static balance task and to maintain this foot position during the entire fitting task. If the subject's hand or the block contacted the sides of the opening or their feet moved, this condition was discarded and the task was repeated. The testing was closely monitored by the experimenters for quality purposes.

During the fitting task, participants were required to fit the block into either a small, medium, or large opening (fitting different openings condition) on the board while maintaining a stand position either an arm's length or 1.3 times an arm's length from the board (reaching different distances condition) (Figure 1). Therefore, there were six conditions as follows: (1) large opening with arm's length; (2) medium opening with arm's length; (3) small opening with arm's length; (4) large opening with 1.3 times arm's length; (5) medium opening with 1.3 times arm's length; and (6) small opening with 1.3 times arm's length. All of the conditions were randomized and executed in consecutive trials until five successful fits were achieved. Experimental operators adjusted the placement of the board based on the shoulder height and the length of the dominant arm before each condition. Otherwise, the table was adjusted to the subject's waist height so the blocks could be comfortably picked up. Each trail was accompanied by 2 short beeps to signal the start and end of the fitting task for participants. After the second beep, the experimenter would take the block and put it back on the basement as soon as possible. To prevent fatigue, there were no time constraints between each of the six conditions, so the participants can take their time to finish the protocol.

### 2.4. Data Analysis

All data were recorded and stored on a PC. Force plate data was used to calculate center of pressure (COP) of foot for all trails. The COP data was low-pass filtered with a Butterworth digital filter of fourth-order and cut-off frequency of 50 Hz. Only a third of the static balance task and a fifth of the fitting task data were processed by Excel (Microsoft, Washington, USA). The posture sway was quantified using maximum displacement in both the anterior-posterior (AP) and mediolateral (ML) directions, the 95% confidence ellipse area (95% area), and the mean velocity. For the static balance task, 30-second COP of foot was analyzed. For the fitting task, we analyzed the total fitting time that was recorded by optical gate sensors of COP for each condition.

### 2.5. Statistical Analysis

Two-way ANOVA with repeated measures was used to identify the association between dependent variables (COP variables) and independent variables (group, vision) for static balance test. Three-way ANOVA with repeated measures was used to identify the association between dependent variables (COP variables) and independent variables (group, size, and distance) for the fitting task. Then significant associations were examined further using univariate analysis and post hoc Tukey's test. All statistical analysis was conducted in SPSS system (19.0, SPSS Inc., Chicago, IL, USA). Significant level was set at 0.05.

## 3. Result

### 3.1. Demographic

Table 1 shows the demographic characteristics for the three groups. No significant group effects were observed.

### 3.2. Maximum Displacement of COP in the AP and ML Direction

**Figure 2:**
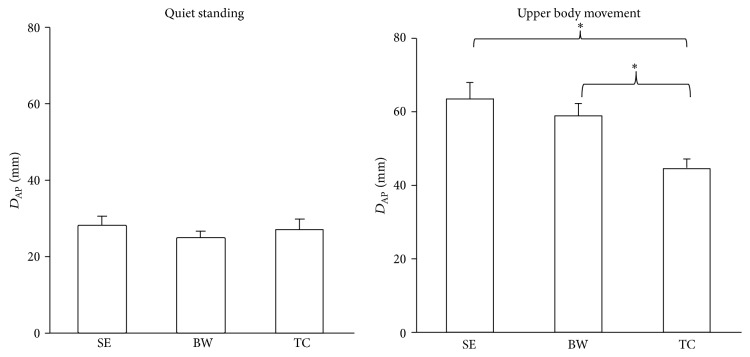
The maximum displacement of COP in the AP direction was collected in the quiet standing and standing with upper body movement. The TC group had better postural control than the SE and BW groups when standing with upper body movement. Values are group means ± SE, with “*∗*” representing significant differences (where TC group were significantly different from the other groups).

**Figure 3:**
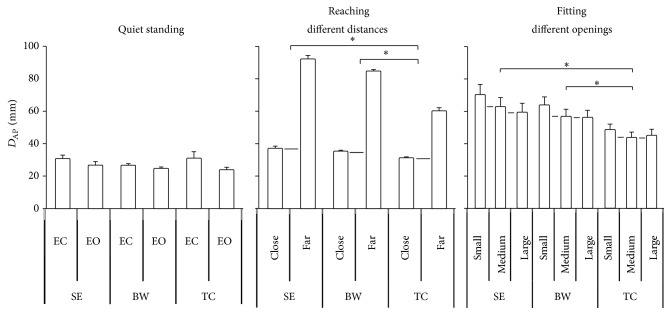
The maximum displacement of COP in the AP direction of quiet standing, reaching different distances, and fitting different openings. The slope of TC group was less than the BW and SE groups. Values are group means ± SE, with “*∗*” representing significant differences (where TC group were significantly different from the other groups for both distances and openings).

**Figure 4:**
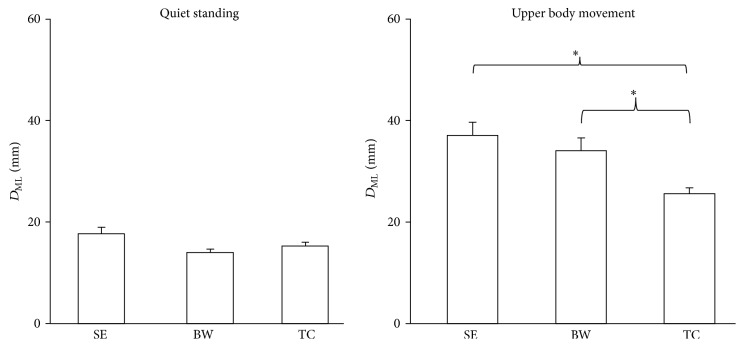
The maximum displacement of COP in the ML direction as collected in the quiet standing and standing with upper body movement. The TC group had better postural control than the SE and BW groups when standing with upper body movement. Values are group means ± SE, with “*∗*” representing significant differences (where TC group were significantly different from the other groups).

**Figure 5:**
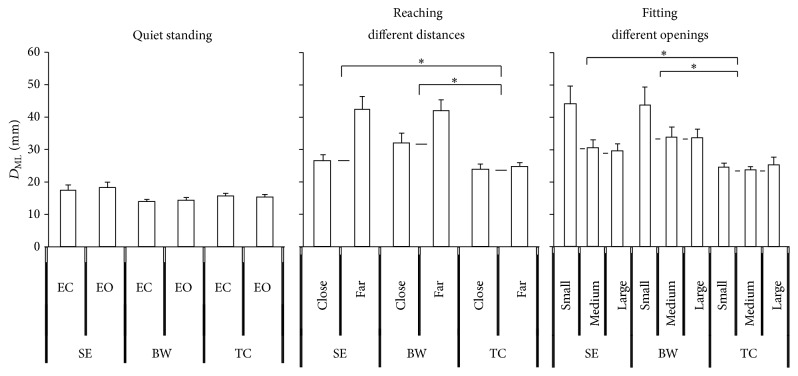
The maximum displacement of COP in the ML direction of quiet standing, reaching different distances, and fitting different openings. The slope of the TC group was less than the BW and SE groups. Values are group means ± SE, with “*∗*” representing significant differences (where TC group were significantly different from the other groups for both distances and openings).

The quiet standing condition showed less displacement than the reaching different distances and fitting different opening conditions for both directions. There were no significant group X vision interactions observed. There were no significance effects for the quiet standing condition between the EC and EO conditions, nor between groups. However, significance effects were observed for the group in the upper body movement condition in the AP direction (*F*_2,33_ = 11.551, *p* < 0.0001) (Figure 2) and ML direction (*F*_2,33_ = 4.170, *p* = 0.024) (Figure 4). The TC group had less maximum displacement in both directions for upper body movement condition than the SE and BW groups. The reaching far distance condition led to greater maximum displacement than the reaching close distance condition in the AP (*F*_1,33_ = 462.072, *p* < 0.0001) (Figure 3) and ML directions (*F*_1,33_ = 22.057, *p* < 0.0001) (Figure 5). Furthermore, the fitting different openings condition had significant effects observed in the AP (*F*_2,33_ = 15.136, *p* < 0.0001) (Figure 3) and ML directions (*F*_2,33_ = 8.044, *p* = 0.003) (Figure 5). The fitting small opening condition created greater maximum displacement than the reaching medium and larger openings condition in both directions. The distance by group also showed statistical significance in the AP (*F*_2,33_ = 14.489, *p* < 0.0001) (Figure 3) and ML directions (*F*_2,33_ = 8.044, *p* = 0.003) (Figure 5). Post hoc testing showed that the TC group had less maximum displacement than the SE and BW groups in both directions. The tests of within subject contrasts showed significant linear trends for the reaching different distances condition (AP: *F*_1,33_ = 462.072, *p* < 0.0001; ML: *F*_1,33_ = 22.057, *p* < 0.0001) and fitting different openings condition (AP: *F*_1,33_ = 23.688, *p* < 0.0001; ML: *F*_1,33_ = 8.044, *p* = 0.003) in both directions (Figures 3 and 5). This indicates increased maximum displacement for increased reaching distance or decreased fitting opening. Also, the tests showed significant quadratic trends for the fitting different openings condition in the ML direction (*F*_1,33_ = 7.010, *p* = 0.012) (Figure 5). The distance by group interaction also showed significant linear trends in AP (*F*_2,33_ = 14.489, *p* < 0.0001) (Figure 3) and ML (*F*_2,33_ = 4.954, *p* = 0.013) (Figure 5) direction which means the slope of maximum displacement of the TC group is less than the SE and BW groups with decreased reaching distance. There was no significant group by distance by opening three-way interaction having been observed.

### 3.3. The 95% Confidence Ellipse Area

**Figure 6:**
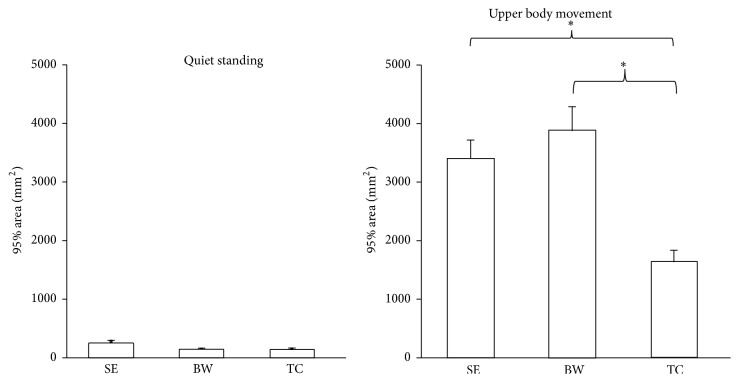
The 95% confidence ellipse area of COP was collected in the quiet standing and standing with upper body movement tests. The TC group had better postural control than the SE and BW groups when standing with upper body movement. Values are group means ± SE, with “*∗*” representing significant differences (where TC group were significantly different from the other groups).

**Figure 7:**
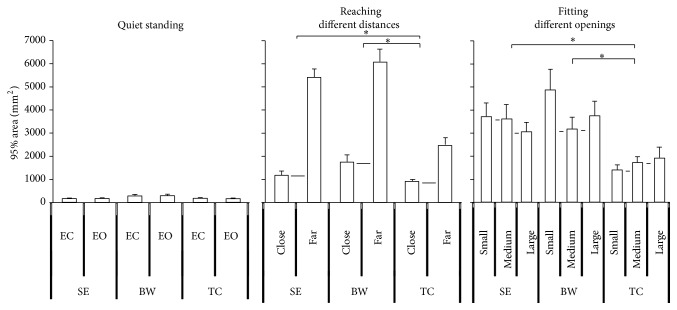
The 95% confidence ellipse area of COP of quiet standing, reaching different distances, and fitting different openings. The slope of TC group was less than BW and SE. Values are group means ± SE, with “*∗*” representing significant differences (where TC group were significantly different from the other groups for both distances and openings).

The result indicated that the quiet standing condition showed a smaller area than the upper body movement condition. In the quiet standing condition, no statistically significant difference was found between the EC and EO conditions. In the upper body movement condition, different groups had significant effects detected on the 95% area (*F*_2,33_ = 10.63, *p* < 0.0001) (Figure 6). The TC group showed less area than the SE and BW groups. The reaching different distances condition also had significant effects detected (*F*_1,33_ = 96.467, *p* = 0.000) (Figure 7). And the distance by group interaction (*F*_2,33_ = 13.643, *p* = 0.003) (Figure 7) was observed for the fitting task. Post hoc test showed that the TC group had less area than the BW group in the fitting close distance condition and had less area than the SE and BW groups in the fitting far distance condition. Otherwise, the tests within subject contrasts showed significant linear trends for reaching different distances (*F*_1,33_ = 96.467, *p* < 0.0001) (Figure 7) where an increase in 95% area was observed for increased distance. In addition, distance by group interaction (*F*_2,33_ = 6.822, *p* = 0.003) (Figure 7) was observed. When the reaching distance was shortened, it was observed that the slope of the TC group was less than the sedentary and BW groups. Furthermore, the tests showed significant quadratic trends for opening by group interaction (*F*_2,33_ = 3.278, *p* = 0.05) (Figure 7) which indicates that increasing the opening size leads to different change between three groups.

### 3.4. Mean Velocity

**Figure 8:**
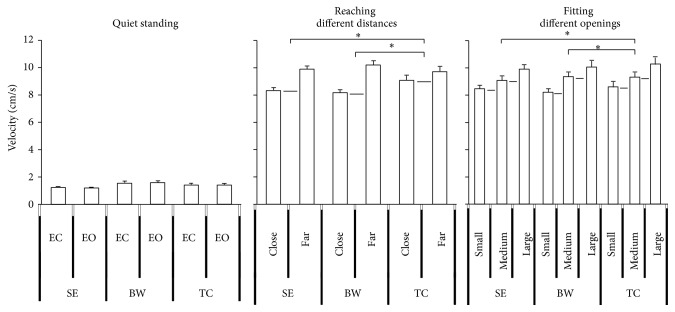
The mean velocity of COP of quiet standing, reaching different distances, and fitting different openings. The slope of TC group was less than BW and SE. Values are group means ± SE, with “*∗*” representing significant differences (where TC group were significantly different from the other groups for both distances and openings).

**Figure 9:**
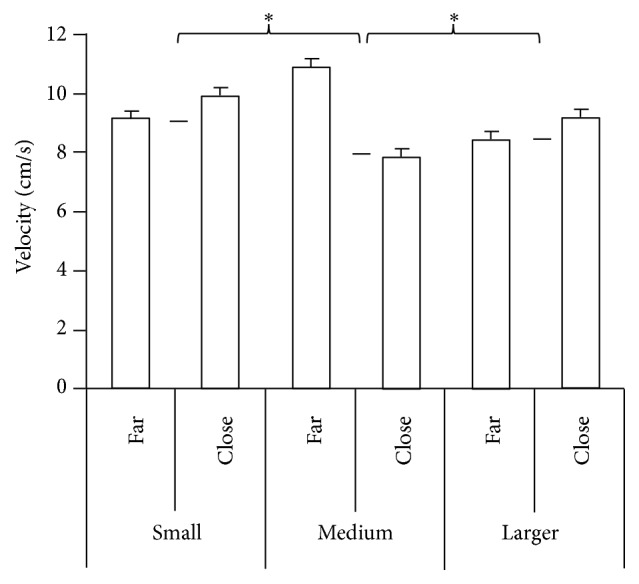
The mean velocity of COP of standing with upper body movement. The distance by opening interaction showed statistical significance. Values are group means ± SE.

The mean velocity for the upper body movement condition was greater than for the quiet standing condition. But, there were no statistically significant differences for the quiet standing condition between the EC and EO conditions. Significant effects were observed for the reaching different distances condition (*F*_1,33_ = 67.585, *p* < 0.0001) (Figure 8) which means an increase in velocity was observed for decreased reaching distance. The fitting different openings also had significant effects (*F*_2,33_ = 41.306, *p* < 0.0001) (Figure 8) with an increase in velocity for an increased opening size. In addition, the distance by group (*F*_2,33_ = 41.306, *p* < 0.0001) (Figure 8) interaction was observed in the fitting task. Post hoc test observations revealed that the TC group demonstrated less velocity than the BW group in close distances. The tests of within subject contrasts showed significant linear trends for reaching different distances (*F*_1,33_ = 67.585, *p* < 0.0001) (Figure 8) which indicates an increase in velocity for decreased distance. Fitting different openings (*F*_1,33_ = 63.961, *p* = 0.000) (Figure 8) also had significance linear effects on velocity with an increase in velocity for larger openings. The distance by group interaction (*F*_2,33_ = 5.716, *p* = 0.007) (Figure 9) showed statistically significance linear trends. The performance of the TC group slopes of the velocity was less than for the SE and BW groups with decreased reaching distance. In addition, the distance by opening by group interaction (*F*_2,33_ = 3.597, *p* = 0.039) also showed significant linear trends which indicates the slope of velocity of the TC group is less than the SE and BW groups with change distances and opening sizes.

## 4. Discussion

The purpose of this study was to examine the effect of TC training on postural control with additional upper limb movement. We found that although regularly practiced TC and BW, as compared with SE, were not effective in improving posture stability during static balance task, the positive influence was observed during fitting task as upper limb movement was required. The current data support our hypothesis.

### 4.1. Quiet Standing Condition

Previous studies demonstrated that visual input [25] and physical activity [19, 20] play an important role for static balance control. For example, Guan observed less COP sway area in the TC group than in the control group under four standing conditions (standing still with eyes open; standing still with eyes closed; standing and turning head to the left and right with eyes open; standing and turning head to the left and right with eyes closed) [19]. In our study, statistical results showed no significant effects among the three groups which means no effect on static balance was detected. Hence, in all of the groups, there was no obvious difference in balance control during quiet standing. Many researchers have demonstrated that the elderly people suffer more risks of falls when performing dual task [2]. In summary, the result of quiet standing condition cannot prove the distinction of postural control among the three groups. In other words, static balance control was consistent with quiet standing.

### 4.2. Reaching Different Distances

Theoretically, as people age, difficult manual tasks have more constraints [26, 27]. The reaching near distance condition encountered less balance perturbations than the reaching far distance condition. All of the outputs supported this standpoint. Further analysis showed that the TC group exhibited less perturbation from a reaching near distance condition than from a far distance condition compared to the BW and SE groups. However, the SE and BW groups demonstrated the same difficulty in the reaching different distances condition.

The result suggests that when the perturbation of trunk movement was more difficult, the TC group had better postural stability than the SE and BW groups. In comparison to the fitting near target, the far target approach focuses on the interaction among hip, ankle, and orientation [28]. Previous studies on fitting tasks have reported evidence in support of movement and modulating strategy for adults and older children [11]. That is, a more robust and adaptable movement and modulating strategy replaced the movement and stabilization strategy for adults. In our study, TC exercise may be more valid to maintain or promote movement and modulating strategy. The possible mechanism may be the hand movement accompanied with weight shifting in TC performance which reduces the perturbation of balance when fitting far target.

### 4.3. Fitting Different Openings

In general, arm movement generates perturbations of balance when upright standing [29]. Otherwise, in the block through the small opening, hand precision of the endpoint becomes important [11]. We observed a small magnitude of deviations of outputs in the fitting different openings condition by the TC group compared with the BW and SE groups. Additionally, there was no difference in stability between the BW and SE groups.

Many researches demonstrated that intervention techniques can improve fine manual performance in older adults [29, 30]. However, BW and SE appear to impact whole body postural functions the same. Bernstein detected that the hammer's trajectories were exhibited without competition when striking a nail; however, the endpoint was competition [31]. He suggested that the release of the redundant degree of freedom was a central issue in motor control. BW exercise is a simple, optionally cyclical movement which means the coordination among the segments was not critically important, whereas TC exercise is coordinated, precise movement. There is variability in the trajectory of the arm movement, but the endpoint is consistent. Haddad et al. speculated that it is easier for young children to freeze the trunk to control hand precision than it is for older children and adults [11]. Therefore, it is possible that the mechanism of TC exercise that reduces redundant degrees of freedom [31] may make standing with reaching different openings easier to control [11].

One limitation of this study is that the design is a cohort study rather than a randomized controlled trail. Therefore, there may be unavoidable selection bias that may interfere with results. Therefore, future study should be designed by randomized controlled trail. Another limitation of this study is that we had not recruited a daily function group: the participants only engaged in long-term housework, such as cooking, sweeping, and folding laundry, without other physical activities. The upper body perturbation should improve the postural control during daily life. Therefore, the daily function group that requires both quiet standing task and fitting task should be tested in future study.

This study observed the postural control of long-term TC, BW, and SE during quiet standing and fitting conditions. With the quantification of COP data during these tasks, we recognized that rhythmic weight shifting with upper body motion might decrease the risk of fall and improve the quality of daily life for older adults. This observation can be used to design balance training where upper extremity can be used as self-generated perturbation in a safe environment for more effective postural control rehabilitation exercise.

## 5. Conclusion

In this study, the result showed that long-term TC practitioners effectively decreased their posture sway during upper body movements comparing to the BE and SE groups. Hence, long-term, regular TC exercise could link to decreased risk of falls for older people. This potential benefit could be due to the fact that TC exercise demands highly accurate movements and the upper limb movements effectively serve as challenges for lower extremity movements during Tai Chi practice. The interaction between upper and lower extremity movements during Tai Chi practice and its benefit for postural control should be further examined in the future using well designed intervention studies.

## Figures and Tables

**Table 1:** Demographic information of three groups (TC: male = 8, female = 4; SE: male = 7, female = 5; BW: male = 8, female = 4).

	Mass (kg)	Height (cm)	BMI (kg/m^2^)
TC	63.43 ± 9.34	162.51 ± 6.87	23.98 ± 2.91
SE	68.28 ± 7.01	163.94 ± 7.01	25.41 ± 2.96
BW	62.78 ± 6.59	163.48 ± 6.36	23.54 ± 2.74

*Note.* Each parameter stands for mean ± standard deviation (M ± SD). TC = Tai Chi; SE = sedentary; BW = Brisk walk.
